# Analysis of factors affecting hospitalization costs in Parkinson disease patients: A retrospective study based on 848 cases

**DOI:** 10.1097/MD.0000000000041540

**Published:** 2025-02-14

**Authors:** Yatan Li, Xiaoqiang Ma, Shanquan Zhang, Yingying Jia, Fan Yang, Wei Jia

**Affiliations:** a Department of Pharmacy, The Second Affiliated Hospital, Xinjiang Medical University, Urumqi, China; b School of Public Health, Xinjiang Medical University, Urumqi, China; c Information Center, The Second Affiliated Hospital, Xinjiang Medical University, Urumqi, China; d Information and Big Data Management Center, Affiliated Cancer Hospital, Xinjiang Medical University, Urumqi, China.

**Keywords:** disease diagnosis, disease treatment, economic burden, hospitalization costs, Parkinson disease

## Abstract

This retrospective study aimed to examine hospitalization costs and associated factors for Parkinson disease (PD) patients. A total of 848 PD patients were extracted from the National Neurological and Brain Center in Xinjiang, and basic demographic information, medical insurance payment, and hospital expenses were collected. SPSS 26.0 was used and univariate analysis was performed on the number of days of hospitalization, times of hospitalizations, gender, payment method, and total hospitalization costs. Correlation analysis was performed using multiple linear regression to explore the influencing factors of hospitalization expenses. The average hospitalization cost of PD patients was 15,515.78 ± 29,667.49 yuan. Diagnosis fees accounted for the highest proportion (29.50%), followed by material costs (25.50%) and drug costs (21.20%). PD patients with less than 5 hospitalizations were the main source of disease, but 2.83% of them had more than 10 hospitalizations (*P* = .025). Female patients were significantly more than male patients, but the total hospitalization costs of male patients were much higher than those of female patients (*P* < .001). The number of PD patients increased significantly when they were over 60 years old, but the hospitalization costs were much lower than those of patients under 60 years old (*P* = .007). The effect of the payment method on total hospitalization cost did not show a statistical difference (*P* = .213). There was a positive correlation between hospitalization days and total hospitalization cost (*P* < .001). In conclusion, the results of the regression model suggested that age, length of hospital stay, gender, and number of hospitalizations could be used as predictive variables for hospitalization costs in PD patients.

## 1. Introduction

Parkinson disease (PD) is a common neurodegenerative disease in the elderly. The current clinical diagnosis still follows the definition of the International Parkinson’s Disease and Movement Disorder Society and is shown as a core clinical motor syndrome (Parkinsonism) accompanied by substantia nigra pars compacta neurodegeneration and synuclein deposition.^[1]^ However, the accumulated evidence in recent years shows that the diagnosis for PD is lagging behind, and diagnostic methods and new diagnostic biomarkers will appear in the next few years. The *Lancet* published a systematic analysis of the PD burden in countries around the world from 1990 to 2016. It mentioned that as the course of PD patients prolongs, the number of risk factors for the disease can increase to 84. The mortality rate is higher than 40% in countries with different levels of sociodemographic index. The disability-adjusted life years in high, medium–high or medium sociodemographic index countries are 34.4% and 50.0%, respectively, indicating that PD brings significant medical burdens in countries with different income, education, and fertility rates.^[2]^ Although a large number of studies reported the increase in the prevalence and disease burden of PD patients at home and abroad, there was a lack of investigation into the economic burden of this population.^[3]^ This study was established on the basis of the National Neuroscience Research Center. After conducting a clinical analysis of comorbid polypharmacy in a large number of PD patients, a systematic investigation of the inpatient medical expenses of 848 PD patients was conducted.^[4]^ This study provided baseline evidence for an in-depth exploration of the direct economic burden of PD patients and related influencing factors.

## 2. Material and methods

### 2.1. Research resources

This was a retrospective analysis of the hospitalization medical records of 848 inpatients with PD in a national neurological and brain center in Xinjiang from 2014 to 2022. According to the main diagnosis code G20.x00, various medical expense data about PD patients were collected, including nursing, diagnosis, surgical expenses, treatment, nonsurgical fees, medication costs, material fees, and others. All PD patients who met the PD diagnostic criteria established by the Movement Disorders and Parkinson’s Disease Group of the Chinese Society of Neurology were included.^[5]^

### 2.2. Data collection

The following information was extracted from the first page of the inpatient medical record: basic information: patient name, gender, age, and medical payment style; medical information: number of hospitalizations, length of stay, date of hospitalization, date of discharge, whether there were complications, whether the patient was critically ill during hospitalization, and outcome; cost information: total hospitalization cost, nursing fee, diagnosis fee, surgical fee, treatment fee, nonsurgical fee, drug fee, material fee, and other fees. To prevent bias, all data of patients diagnosed with PD were included in the analysis.

### 2.3. Statistical analysis

SPSS 26.0 was used to conduct a descriptive analysis of the basic information and the medical payment methods of PD patients, as well as the annual sum of the total expenses, nursing fees, diagnosis fees, and surgical fees during the period 2014–2022, were counted. The total hospitalization expenses were normally distributed after the Kolmogorov–Smirnov test and were described by mean and standard deviation. The variables of hospitalization frequency, gender, length of hospital stay, payment method, and total hospitalization cost were analyzed by 1-way ANOVA to explore the factors affecting the economic burden. *P* < .05 was considered statistically significant.

### 2.4. Ethics approval

This study was approved by the Ethics Committee of the Second Affiliated Hospital of Xinjiang Medical University (approval number: IAUCU-20230228-31).

## 3. Results

### 3.1. Demographic characteristics of PD patients

A total of 848 PD patients were admitted from 2014 to 2022. The number of hospitalizations for each patient ranged from 1 to 15 times, of which 563 cases (66.39%) were first hospitalizations, 46 cases (5.43%) were hospitalized 6 to 10 times, and 2.83% of patients were hospitalized more than 10 times. There were 391 male patients (46.10%) and 457 female patients (53.90%). The number of PD patients over the age of 60 has increased significantly. The medical insurance of patients was mainly urban employee medical insurance (61.44%) and urban resident medical one (20.17%). About 11.79% of patients were fully self-funded hospitalizations, and 1.65% of patients used commercial medical insurance (Table 1).

**Table 1:** Results of 1-way analysis of variables and total hospitalization costs.

Variant	Groups	n	Mean ± standard deviation (yuan)	*F*	*P*
Number of hospitalizations	0–5 times	778	16,345.87 ± 30,783.08	3.71	.025
	6–10 times	46	6222.16 ± 6172.74		
	More than 10 times	24	6419.62 ± 6602.5		
Genders	Female	457	12,294.25 ± 19,140.98	11.83	.001
	Male	391	19,281.09 ± 38,167.7		
Age	<60	174	21,239.94 ± 43,638.054	4.06	.007
	60–69	256	16,827.07 ± 34,500.31		
	70–79	227	13,006.45 ± 19,402.01		
	≥80	191	11,525.81 ± 9417.10		
Payment method	Basic medical insurance	706	16,095.32 ± 29,471.14	1.50	.213
	Commercial medical insurance	1	7718.66 ± 0.00		
	Other medical insurance	40	19,384.41 ± 46,406.95		
	Self-financed	101	10,009.75 ± 21,265.03		
Days of hospitalization	1–10 d	267	9951.92 ± 14,237.61	32.64	.001
	11–20 d	478	14,325.76 ± 23,474.31		
	21–30 d	98	31,764.81 ± 55,076.94		
	≥31 d	5	107,910.3 ± 119,352.49		

### 3.2. Composition characteristics of hospitalization expenses of PD patients

**Figure 1.**
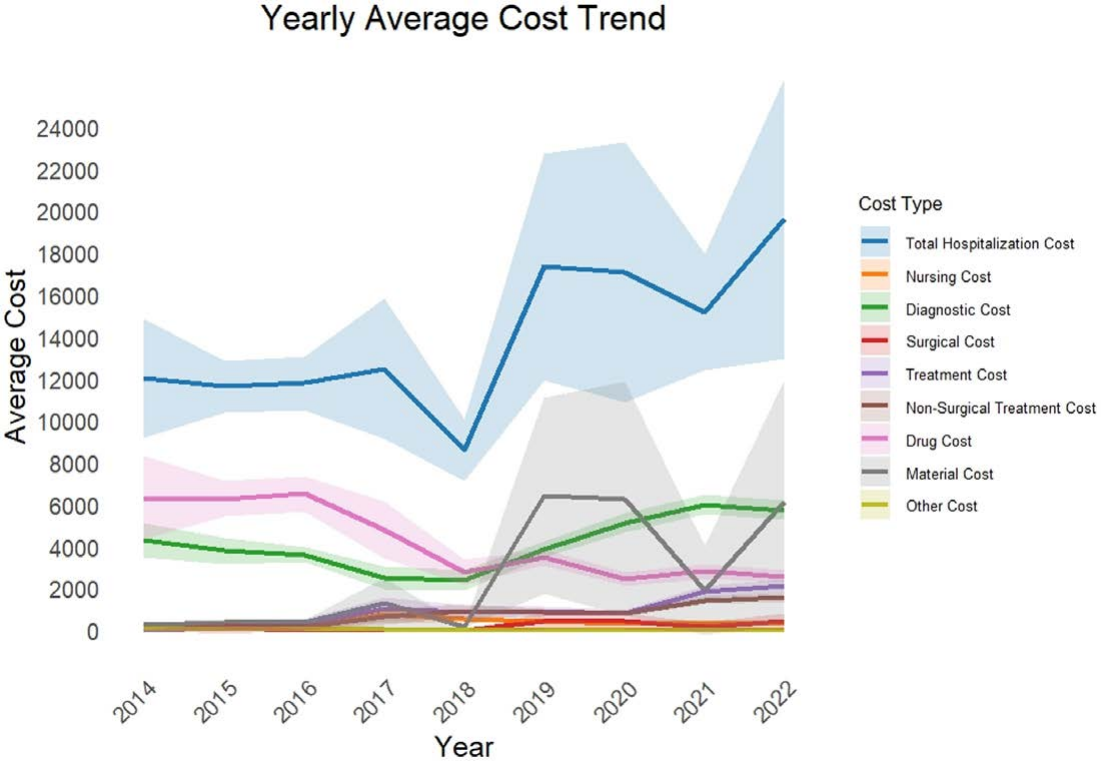
Changing trend of annual hospitalization expenses of PD patients. PD = Parkinson disease.

Previous studies have shown that PD patients often suffer from multiple comorbidities, and each patient takes a different amount of therapeutic medication.^[4]^ From an economic perspective, this survey reflected the phenomenon that PD patients had higher hospitalization costs to varying degrees. During this investigation, the total hospitalization costs of PD patients in all were 787,764.45 yuan, with higher fees for patients in 2019 and 2020 (Fig. 1). Among the various medical expenses between 2014 and 2022, the average hospitalization cost was 15,515.78 ± 29,667.49. Diagnosis fees accounted for the highest proportion of hospitalization expenses for PD patients (29.50%), followed by material fees and drug fees, which were 25.50% and 21.20%, respectively (Table 2).

**Table 2:** Composition ratio of annual average hospitalization expenses of PD patients.

Cost category	Mean ± standard deviation (yuan)	Constituent ratio (%)
Total hospital expenses	15,515.78 ± 29,667.50	100.00%
Nursing fee	386.38 ± 657.80	2.50%
Diagnostic fee	4576.02 ± 2882.53	29.50%
Operation fee	291.48 ± 1916.44	1.80%
Treatment expense	1223.39 ± 1700.00	7.90%
nonsurgical treatment fee	1029.74 ± 1261.37	6.60%
Pharmaceutical expenses	3280.03 ± 2853.71	21.20%
Cost of materials	3963.05 ± 25,532.96	25.50%
Other expenses	25.84 ± 34.08	0.16%

PD = Parkinson disease.

### 3.3. Univariate analysis of various information on PD patients

The 1-way analysis of variance was used to verify the number of hospitalizations, age, gender, payment method, and total hospitalization costs. The results showed that there was a statistically significant relationship between the number of hospitalizations, age, gender, and total hospitalization costs (*P* < .05), while the relationship between the total hospitalization costs of different payment methods was not statistically significant, and it could not be considered that there was a difference. The total hospitalization cost followed a normal distribution by Kolmogorov–Smirnov test (*P* > .05), so it was described as mean ± standard deviation (Table 1).

PD patients with less than 5 hospitalizations were the main source of disease, but 2.83% of patients had more than 10 hospitalizations (*P* = .025). This is enough to treat PD as a long clinical process. An interesting point is that there are significantly more female patients than male patients, but the total hospitalization costs of male patients are much higher than those of female patients (*P* < .001). However, why there is such a difference is worth further studying from multiple factors of the patients themselves. The number of PD patients increased significantly when they were over 60 years old, but the hospitalization costs were much lower than those of patients under 60 years old (*P* = .007). The effect of the payment method on total hospitalization costs did not show a statistical difference (*P* = .213). The number of hospitalization days for PD patients was concentrated between 11 and 20 days. From the overall trend, there was a positive correlation between the number of hospitalization days and the total hospitalization costs (*P* < .001). The longer the hospitalization days, the more total hospitalization costs. Of course, this is definitely related to the severity of the patient’s disease. Further stratified analysis is needed to prove this point.

### 3.4. Linear regression model

Incorporating the indicators of significant differences into the regression model, we could see that when the number of days of hospitalization, age, gender, and number of hospitalizations were used as predictive variables, they had a significant impact on the total cost of hospitalization. The *R*^2^ = 0.1 in this model indicated that these variables could explain 10% of the total variation in the total cost of hospitalization. The results of the analysis of variance analysis showed that *F* = 37.46, *P* = .000, which confirmed that the model as a whole had a significant predictive ability for the total cost of hospitalization (Tables 3 and 4).

**Table 3:** Goodness of fit for multiple linear regression model.

	*R*	*R* ^2^	Adjusted *R*^2^	Standard error of estimate
Model	0.389*	0.151	0.147	27,401.57

* Predictors: (constant), age, length of hospital stay, gender, times of hospitalizations.

**Table 4:** ANOVA analysis for total hospitalization costs.

Model	Sum of squares	*df*	Mean square	*F*	Significance
Regression	112,532,551,581.77	4	28,133,137,895.44	37.46	0.000†
Residual	632,963,209,880.12	843	750,846,037.81		
Total	745,495,761,461.89	847			

ANOVA = analysis of variance.

*Dependent variable: total hospitalization costs.

† Predictors: (constant), length of hospital stay, gender, age, number of hospitalizations.

### 3.5. Linear regression analysis of factors affecting the total hospitalization cost of PD patients

The *β* of the number of hospitalizations was −1567.53, which had a significant negative impact on the total hospitalization costs (*P* < .001). This result may indicate that as the number of hospitalizations increased, the total hospitalization costs of patients decreased. This phenomenon may be related to the more standardized treatment plans and more effective disease management of patients with multiple hospitalizations, resulting in relatively low single-hospitalization costs. In addition, it might reflect the cost–control strategy of medical institutions for patients with repeat hospitalizations or the economic considerations of patients regarding treatment options.

The *β* value for gender was −7247.02, indicating that the total hospitalization cost of male patients was significantly higher than that of female patients (*P* < .001). This might be related to the differences between men and women in the disease spectrum, choice of hospitalization treatment, and medical needs. High-cost diseases or treatments might be more common in men, and male patients were more likely to receive higher-level and more expensive medical services. Such results might also be related to the physical conditions of patients of different genders.^[6]^

*β* = −198.97 for age, it had a negative impact on total hospitalization costs. This finding might indicate that as age increases, the need for nursing services gradually decreases, and elderly patients might be more inclined to choose conservative treatment, rather than more invasive and costly treatments. The reason for this might be that older patients were in weaker health and could not tolerate complex surgeries or expensive treatments. Elderly patients might enjoy more comprehensive medical insurance coverage, which makes their actual hospitalization cost burden lower.

The *β* of the length of hospital stay was 1767.43, indicating that there was a strong positive correlation between the length of hospital stay and the total cost of hospitalization (*P* < .001). This result was in line with expectations because longer hospital stays mean more demand for treatment, examinations, and nursing services, which led to an increase in total costs. The significance and size of this coefficient emphasized that the length of hospital stay was a key factor in cost prediction and was of great significance to the management and control of medical expenses (Table 5).

**Table 5:** Linear regression analysis of the impact of total hospital expenses.

Model	Unstandardized coefficients	*B*	*t*	Siginificance	Standardized coefficients
	(Constant)	12,489.94	1.86	0.017	
	Number of hospitalizations	−1567.53	−3.66	<0.001	−0.12
	Gender	−7247.02	−3.68	<0.001	−0.12
	Length of hospital stay	1767.43	11.04	<0.001	0.36
	Age	−198.97	−2.15	0.003	−0.07

*Dependent variable: total hospitalization costs.

## 4. Discussion

### 4.1. Economic burden of PD patients

The results of the hospitalization cost analysis of 848 PD patients showed that the average annual hospitalization cost of patients was 15,515.78 yuan, the out-of-pocket amount was 9998.76 yuan, and the medical insurance reimbursement rate was about 30%. There is no cure for PD yet, which is the reason why most PD patients need multiple hospitalizations and long-term use of therapeutic drugs and assistive devices. Although our country has a high medical insurance coverage rate, this part of medical expenses is still 1 of the economic burdens of many families.^[7]^ However, whether this part of the disease burden has caused great difficulties for the patients themselves should be 1 of the directions of the next patient survey.

### 4.2. Effect of hospital stay on hospitalization costs of PD patients

The number of days of hospitalization had a strong positive correlation with the total hospitalization costs, which was mainly reflected in the following aspects. Direct medical expenses: the longer the length of hospital stay, the higher the direct medical expenses, including drug costs, medical equipment usage fees, nursing costs, and examination costs. Treatment and care costs: long-term hospitalization might require more treatment and care interventions, including medication adjustments, complex medical procedures, and long-term monitoring, which could increase medical costs. Risk of complications: patients who were hospitalized for a long time were more likely to develop complications such as hospital-acquired infection, pulmonary embolism, and pressure sores. Treating these complications could increase additional medical expenses. Rehabilitation costs: PD patients with longer hospitalization time might need more rehabilitation treatment to improve motor function and quality of life, which also increases hospitalization costs. As the length of hospital stay increased, the above costs accumulated and gradually increased, thus leading to an increase in the total costs in this study. There was no definitive conclusion on the etiology and development of PD at home and abroad, which had a profound impact on the rapid and effective outcome of patients.^[8]^ Therefore, clinicians need to follow the existing diagnosis and treatment standards and screen the RCT results and multicenter efficacy and safety clinical trials in recent years on PD clinical research.^[9]^ Then, under the premise of ensuring the quality of medical care as much as possible, we should optimize medical care and shorten the length of hospital stay, thereby reducing the corresponding medical expenses and alleviating the financial pressure on patients. In addition, we should vigorously carry out scientific research projects on PD patients over time to find treatment methods or therapeutic drugs as soon as possible.

### 4.3. The impact of the number of hospitalizations on the total cost of hospitalization

As its chronic development characteristics, PD patients might be hospitalized multiple times, so the number of hospitalizations was also one of the factors affecting the total hospitalization costs. After data analysis, it was found that the number of hospitalizations had a negative impact on the total hospitalization costs. This was reflected in the fact that the more hospitalizations, the lower the total hospitalization costs could be compared with the previous total hospitalization costs. This may be because factors such as optimized medical services, continuously improved treatment plans, and individual medication can reduce the progression of neurodegeneration.^[10]^

### 4.4. Effect of age on total hospitalization costs

Age also has a series of effects on the total hospitalization costs of PD patients.^[11]^ In this research, elderly patients might receive more conservative treatments, or due to their older age, they might not be suitable for certain high-cost treatments or surgeries, resulting in lower hospitalization costs. Elderly patients may have shorter hospital stays because they may be more inclined to receive outpatient treatment or transfer to long-term care facilities rather than long-term hospitalization. For the aspect of medical insurance and expense reimbursement, elderly patients may enjoy more medical insurance and reimbursement policies, resulting in lower actual hospitalization costs. This part of the conclusion can actually be further demonstrated through patient questionnaires.^[12]^

### 4.5. Measures to reduce hospitalization expenses of PD patients

In terms of cost control management for PD patients, we attempt to shorten hospital stays by improving the efficiency of diagnosis and treatment.^[13]^ Using accurate diagnostic tools and effective treatment plans can accelerate the control and improvement of the disease. At present, in addition to the diagnosis and treatment methods of lifestyle, blood biochemical indicators, and skin biopsy, clinicians are also studying the prodromal symptom data model, genetic characteristics, and digital biomarkers obtained from the recent trial of wearable sports trackers.^[14]^ Vigorously promoting multidisciplinary team management is an effective way to ensure the prognosis of various diseases.^[15]^ Establishing a multidisciplinary team including neurologists, nurses, physical therapists, and nutritionists to provide comprehensive treatment.^[16]^ This teamwork can improve the treatment effect, reduce complications, and thus shorten the hospital stay. Optimize the drug treatment plan: use drugs rationally, avoid overtreatment or unnecessary treatment, and reduce rehospitalization due to drug side effects. But in fact, this work is still a long way to go, because anti-PD drugs are still on the road of exploration.^[17]^ Use health information technology: use electronic health records, remote monitoring, and other technical means to monitor patients’ conditions in real time, adjust treatment plans in a timely manner, and reduce acute exacerbations and hospitalizations.^[18]^ Develop personalized treatment plans: considering the differences in the conditions of PD patients, developing personalized treatment plans can effectively control the condition and avoid unnecessary medical interventions and hospitalizations. Optimize the diagnosis and treatment process: shorten the length of hospital stay by improving the efficiency of diagnosis and treatment. The use of accurate diagnostic tools and effective treatment plans can speed up the control and improvement of the condition. The government and medical institutions can consider the special needs of elderly patients and develop more reasonable medical insurance and support policies to ensure that they can obtain necessary medical services. We need to develop personalized medical and nursing plans for elderly patients to ensure that they receive appropriate treatment and care without increasing their financial burden. We also need to strengthen health education and preventive measures for elderly patients and their families to help them better manage PD and reduce unnecessary hospitalization needs.

### 4.6. Strengths and limitations

This study provided baseline evidence for an in-depth exploration of the direct economic burden of PD patients and related influencing factors based on 848 PD patients in the National Neurological and Brain Center in Xinjiang. The results showed an information for us that age, length of hospital stay, gender, and number of hospitalizations could be used as predictive variables for hospitalization costs in PD patients. Meanwhile, these factors were also the key to ameliorating the hospitalization costs of PD patients.

Although this study was based on a relatively sufficient data sample size, it was still a single-center study. We will use this as a baseline to establish a simultaneous study in multiple medical alliance hospitals. Multicenter research can verify the results of our study and provide help for jointly optimizing the medical expenses of PD patients in the future, providing a more accurate basis for reducing medical expenses for patients with PD.

## Author contributions

**Funding acquisition:** Yatan Li.

**Investigation:** Yatan Li.

**Formal analysis:** Xiaoqiang Ma, Shanquan Zhang.

**Software:** Xiaoqiang Ma.

**Resources:** Yingying Jia.

**Writing – original draft:** Fan Yang, Wei Jia.

**Writing – review & editing:** Wei Jia.
